# Audit of the use of the physical health improvement (PHIT) to document physical health examination on an electronic health record at a mental health trust in Manchester

**DOI:** 10.1192/bjo.2021.77

**Published:** 2021-06-18

**Authors:** Anthony Baynham

**Affiliations:** Greater Manchester Mental Health NHS Foundation Trust

## Abstract

**Aims:**

The audit aimed to identify: The percentage of patients with Initial Physical Examination (IPE), ECG and bloods on admission being completed; If IPE, bloods and ECG result are documented on PHIT; To identify reasons for these interventions not being completed and review if refusal is being appropriately documented.

**Background:**

“The Five Year Forward View for Mental Health NHS” report highlighted the poor physical health of those with mental health problems when compared to those without. In order to improve the identification and treatment of physical health problems within mental health inpatients, blood test results, physical examination and ECG results should be recorded and reviewed regularly. Within Greater Manchester Mental Health trust, the electronic records system PARIS contains a specific care document to record physical health interventions, known as the PHIT tool. The inpatient unit Park House, had recently changed to the PARIS system prior to this audit and the use of PHIT tool to monitor physical health parameters was considered a priority by the management team.

**Method:**

All admissions to Park House inpatient unit, Manchester in April 2019 were audited. Patients were identified using a report prepared by Business Intelligence. Electronic notes were reviewed for evidence of physical interventions on admission and input of these data to the PHIT tool. Using a retrospective review of electronic notes, relevant information was anonymised and collected to a spreadsheet for further analysis. Inclusion/exclusion criteria was based on local conditions and practical consideration.

**Result:**

An initial sample of 140 was reduced to 89 patients following application of inclusion/exclusion criteria. Of the 89 patients included, 73% had an IPE, 84% of patients had admission blood tests and 74% had an admission ECG. Recording of parameters on the PHIT tool was lower than expected with information recorded in 33–42% of patients. Where patients had refused IPE, ECG or bloods, a valid reason for refusal was documented between 63–91% of patients.

**Conclusion:**

The initial audit identified that most patients had IPE, ECG and bloods but this was documented appropriately in less than 42% had this appropriately documented.

Interventions to improve this rate were developed, focussing on increasing completion of IPE, ECG and bloods as well as improving documentation. The completion of PHIT document is now monitored regularly. The re-audit to identify the magnitude of improvements from these interventions is currently underway.

